# Current trends of high-risk gene Cul3 in neurodevelopmental disorders

**DOI:** 10.3389/fpsyt.2023.1215110

**Published:** 2023-07-28

**Authors:** Ping Lin, Jie Yang, Shumin Wu, Tong Ye, Wenting Zhuang, Wei Wang, Tao Tan

**Affiliations:** ^1^Oujiang Laboratory (Zhejiang Lab for Regenerative Medicine, Vision and Brain Health), Key Laboratory of Alzheimer's Disease of Zhejiang Province, Institute of Aging, Wenzhou Medical University, Wenzhou, Zhejiang, China; ^2^Department of Neuroscience, Baylor College of Medicine, Houston, TX, United States

**Keywords:** Cul3, Cullin3-RING E3 ubiquitin ligases, neurodevelopmental disorders (NDDs), autism spectrum disorder (ASD), knockout (KO) mice

## Abstract

Cul3 encodes Cullin-3, a core component of the ubiquitin E3 ligase that is involved in protein ubiquitination. Recent studies have identified Cul3 as a high-confidence risk gene in neurodevelopmental disorders (NDDs), especially autism spectrum disorder (ASD). Different strategies have been used to generate animal models with Cul3 deficiency in the central nervous system, including whole-brain knockout (KO), cell-type specific conditional KO (cKO), and brain region-specific knockdown. In this review, we revisited the basic properties of CUL3 and its function under physiological and pathological conditions. Recent clinical studies including case reports and large cohort sequencing studies related to CUl3 in NDDs have been summarized. Moreover, we characterized the behavioral, electrophysiological, and molecular changes in newly developed Cul3 deficiency models. This would guide further studies related to Cul3 in CNS and provide potential therapeutic targets for Cul3-deficiency-induced NDDs, including ASD.

## 1. Introduction

Neurodevelopmental disorders (NDDs) are a group of diseases that affect brain development and function. Such disorders include autism spectrum disorder (ASD), developmental delay (DD), intellectual disability (ID), and attention deficit hyperactivity disorder (ADHD) (1, 2). NDDs represent a significant public health concern in society, affecting roughly 1–2.5% of children worldwide (3, 4).

ASD, an NDD characterized by social impairment, stereotyped behavior, and delayed language development (5), has seen a dramatic increase in prevalence over the last few years, reaching 1 to 1.5% in several countries (6), thus becoming a global public health problem. Despite extensive research, the etiology and pathogenesis of ASD remain elusive, with genetic (7) and environmental factors believed to play a critical role in the risk of developing ASD.

Notably, various studies have identified Cullin3 (Cul3) as a high-risk gene for ASD (8, 9). The protein encoded by the Cul3 gene is a core component and scaffold protein of the E3 ubiquitin ligase complex, which plays a crucial role in the ubiquitination and subsequent degradation of specific protein substrates (10).

In this review, we aim to provide a comprehensive explanation of the functional architecture of Cul3 and its physiological and pathological roles in the brain. Additionally, we will summarize recent clinical studies linked to Cul3 in NDDs. Furthermore, we will discuss in detail the characteristics of newly generated Cul3-insufficiency models and novel molecular targets.

## 2. Cullin3-RING ubiquitin ligases

**Figure 1:**
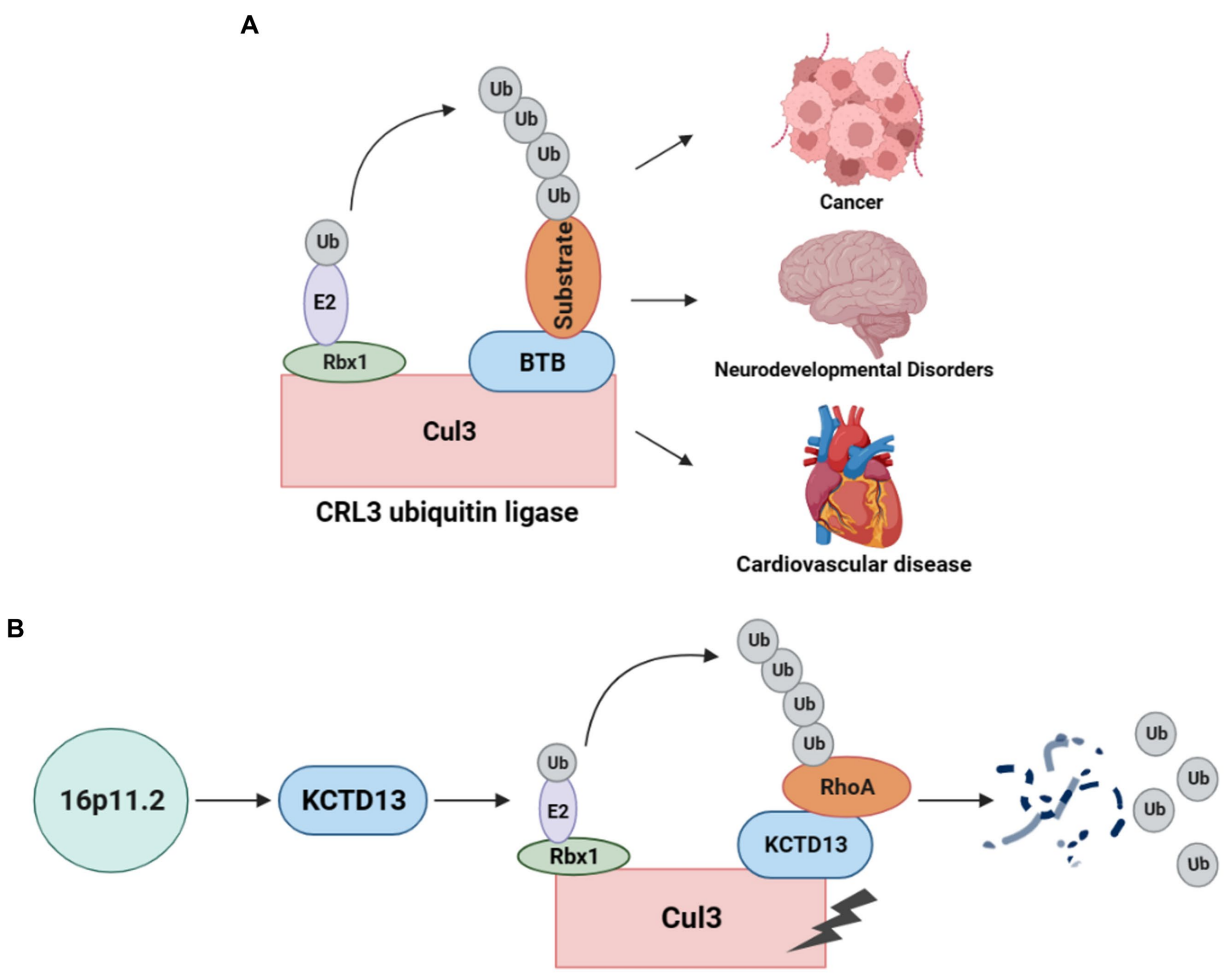
CRL3 ubiquitin ligase complex and its role in animal models of 16p11.2 deletion or duplication. **(A)** A typical CRL3 ubiquitin ligase complex comprises three subunits, including Cul3, Rbx1, and BTB proteins. Cul3 serves as the core component of the complex, which interacts with Rbx1 and E2 at the C-terminus to facilitate the transfer of ubiquitin from E2 to the substrate. At the N-terminus, Cul3 interacts with various BTB proteins, which recognizes and recruits different substrates for ubiquitination and degradation. This complex plays a crucial role in several diseases, such as cancers, neurodevelopmental disorders, and cardiovascular diseases. **(B)** Deletion or duplication of the 16p11.2 CNV leads to a decrease or increase in KCTD13 levels, respectively. The KCTD13-Cul3-RhoA pathway regulates RhoA ubiquitination and degradation.

As a post-translational modification, the ubiquitin-proteasome system (UPS) is responsible for degrading unwanted proteins in eukaryotic cells (11). This process requires a series of enzymes, within which E3 ubiquitin ligase is one of the integral parts of substrate recognition. Among different types of E3 ubiquitin ligases, the Cullin-RING E3 ubiquitin ligases (CRLs) are the most common type (10). Especially, the typical Cullin3 (from the Cul3 gene)-RING E3 ubiquitin ligase (CRL3) complex (Figure 1A) comprises Cul3 protein, RING-box protein 1 (Rbx1), and the substrate recognition subunit Bric-a-brac/Tramtrack/Broad (BTB) protein (12). In the CRL3 ubiquitin ligase, Cul3 does not directly interact with substrate proteins but recognizes and recruits them for ubiquitination through proteins containing BTB domains (13, 14). Rbx1 facilitates the interaction between Cul3 and E2, while Cul3 binds to the substrate proteins through BTB, facilitating the transfer of ubiquitin from E2 to the target protein for degradation. CRL3 ubiquitin ligases are involved in the ubiquitination of various proteins and play crucial roles in several diseases (Figure 1A), including cancer (15), NDDs (16), and cardiovascular diseases (17).

The CRL3 complex was found to involve in ASD with 16p11.2 deletion or duplication (18). KCTD13, located at the 16p11.2 gene locus, is a substrate-recognizing BTB domain adaptor protein that complexes with Cul3 and targets the small GTPase RhoA for ubiquitination and degradation (19) (Figure 1B). Cul3 and KCTD13 proteins physically interact in the middle and late stages of brain development (18), which is critical for regulating the levels of RhoA that control actin cytoskeletal architecture and cell motility (20). This captured significant attention, prompting a surge in studies investigating the role of Cul3 in ASD and NDDs.

## 3. Cul3 expression in the central nervous system

Previous research has highlighted the crucial role of Cul3 in diseases such as breast (21) and ovarian cancer (22). However, recent studies have demonstrated its involvement in central nervous system (CNS) diseases, particularly (NDDs) (23–26).

**Figure 2:**
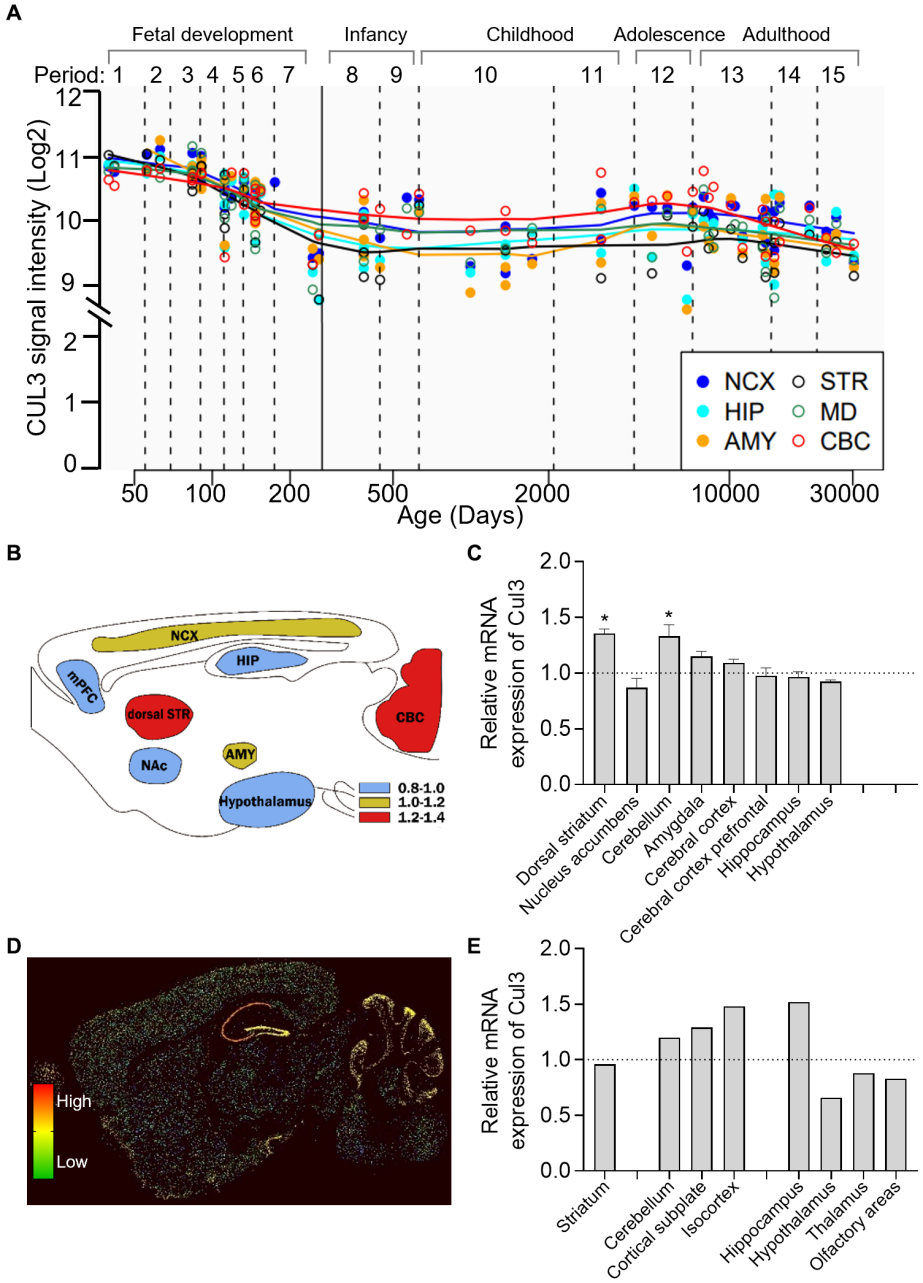
Brain-wide expression of CUL3 in humans and mice. **(A)** Plots depicting CUL3 expression in different regions of the human brain during fetal development (period 1–7), early (period 8–9), young adulthood (period 10–11), adolescence (period 12), and adulthood (period 13–15). Regions include neocortex (NCX), hippocampus (HIP), amygdala (AMY), striatum (STR), medial dorsal thalamus (MD), and cerebellar cortex (CBC). Data obtained from the human brain transcriptome dataset (https://hbatlas.org/, search gene CUL3). **(B,C)** Schematic **(B)** and bar graph **(C)** of Cul3 expression in different mouse brain regions using high-throughput gene expression profiling. Data obtained from bioGPS (http://ds.biogps.org/, search gene Cul3). One–Way ANOVA with Holm-Sidak multiple comparisons test compared to the averaged whole-brain expression, ^*^ *p* < 0.05. **(D,E)** Representative sagittal sections **(D)** and bar graph **(E)** of *in-situ* hybridization of Cul3 mRNA signals in mouse brains. Data obtained from the Allen Institute ISH database (https://mouse.brain-map.org/, search gene Cul3, experiment 75774648).

To investigate the longitude distribution of CUL3 expression in the human CNS, we analyzed the human brain transcriptome (HBT) dataset based (https://hbatlas.org/, search for gene CUL3) (27). As illustrated in Figure 2A, during early embryonic development, CUL3 transcript levels are relatively high in all tested brain regions, including the neocortex (NCX), hippocampus (HIP), amygdala (AMY), striatum (STR), mediodorsal nucleus of the thalamus (MD), and cerebellar cortex (CBC). However, CUL3 transcript levels gradually decrease from late fetal development to infancy (stages 7 and 8), and then remain stable throughout childhood (stage 10). Interestingly, CUL3 transcript levels slightly increase from childhood (stage 11) to adolescence (stage 12) and then decreased again in adulthood. These findings suggest that CUL3 plays a vital role during fetal development and maturation, as showing its highest expression during embryo and adolescent stages. Notably, the transcript levels of CUL3 in CBC and NCX were higher, but relatively lower in STR and AMY compared to other brain regions.

To explore the transcription level of the Cul3 gene in mouse brains, we obtained mouse whole-brain gene expression profiling data from bioGPS (http://ds.biogps.org/, search for gene Cul3). A corresponding expression schematic as shown in Figure 2B. Further analysis revealed that Cul3 showed higher expression levels in the dorsal striatum (*p* < 0.05) and cerebellum (*p* < 0.05) compared to the averaged whole-brain expression (Figure 2C, One-Way ANOVA with Holm-Sidak multiple comparisons tests, *F* (8,36) = 8.954, *p* < 0.0001). In contrast, relatively lower Cul3 expressions were found in the hypothalamus and nucleus accumbens, but without statistical significance. Other brain regions showed averaged expression including the amygdala, cerebral cortex, and hippocampus.

To further explore the spatial distribution of Cul3, we obtained mouse in *in-situ* hybridization (ISH) data from the Allen Brain Atlas (https://mouse.brain-map.org/, search for gene Cul3). Only one experiment was found (75774648, Figures 2D,E) and statistical analysis cannot perform.

Consistent with the high-throughput gene expression profiling data, the ISH data showed high expression levels of Cul3 in the cerebellum and cortex, including the cortical subplate and isocortex (Figure 2E). However, the ISH data showed a contrasting finding in the hippocampus, with significantly high expression levels observed. Moreover, layer-specific distribution was also found, and significantly higher expression levels in the pyramidal and granule cell layers of CA1 and DG of the hippocampus, and the Purkinje layer of the cerebellum (Figure 2D).

In light of these findings, it suggests time-dependent expression, region, and layer-specific distribution of Cul3 in the central nervous system of different species, which warrant further investigation to better understand Cul3 in NDDs.

The PubMed database was utilized to conduct literature search for articles investigating the involvement of Cul3 in the etiology and disease progression of NDDs from January 1st, 2005 to April 30^th^, 2023. The search terms employed were [(Cul3) OR (Cullin3)] and {[(NDD) OR (neurodevelopmental disorder)] OR [(ASD) OR (autism spectrum disorder)] OR [(DD) OR (developmental delay)] OR [(ID) OR (intellectual disability)] OR [(SCZ) OR (schizophrenia)]}. Only publications in the English language were included in this review.

## 4. Clinical studies of Cul3 mutations in NDDs

The genetic architecture of NDDs is complex, involving both rare and common genetic variants. Recent large-scale genetic screening efforts have revealed the significance of loss-of-function mutations of CUL3 in a sizable group of patients with NDDs (Table 1).

**Table 1:** Clinical studies of CUL3 mutations in NDDs.

Ref	Setting	Case	Mutation	Phenotype characteristic
Nakashima et al. (28)	Case-report	3	One missense and two frameshift variants	Global DD, with or without epilepsy.
Iwafuchi et al. (29)	Case-report	1	*De novo* mutations	ASD: patient presented with febrile status epilepticus and developmental regression.
Kato et al. (16)	Case-report	1	*De novo* heterozygous missense variant	DD: ID, macrocephaly, distinctive facial features, and cutis marmorata.
Vincent and Bourque (30)	Case-report	1	A *de novo* splice site variant	Global DD with significant delays in gross motor and language development.
O'Roak et al. (9)	Case–control	677	*De novo* mutations	ASD
De Rubeis et al. (32)	Case–control	3,871	*De novo* LoF mutations	ASD
Hormozdiari et al. (8)	Case–control	1,116	*De novo* mutations	ASD and ID
Codina-Solà et al. (31)	Case–control	36	*De novo* mutations	ASD
Sanders et al. (33)	Case–control	10,220	*De novo* CNVs	ASD
Wang et al. (39)	Case–control	1,543	*De novo* and likely gene-disruptive mutations	ASD
Stessman et al. (34)	Case–control	>14,579	*De novo* mutations	NDDs
da Silva Montenegro et al. (35)	Case–control	>20,000	*De novo* mutations	NDDs
Zhou et al. (36)	Case–control	42,607	*De novo* and inherited LoF variants	ASD
Trost et al. (37)	Case–control	11,312	rare variants	ASD
Wang et al. (38)	Case–control	46,612	*De novo* mutations	ASD and DD

ASD, autism spectrum disorder; NDDs, neurodevelopmental disorders; DD, developmental delay; ID, intellectual disability; LoF, loss of function.

### 4.1. Case-report

Till today, there are six clinical cases (Table 1) that have reported abnormalities resulting from CUL3 mutations, with developmental delay (DD) as the primary phenotype. Nakashima et al. (28) first reported three cases of *de novo* CUL3 variants resulting in global DD, with or without epilepsy. Two of the patients had infantile spasms, while the other had short stature with mild to severe intellectual disability. Whole-exome sequencing identified one missense variant (c.854 T > C, p.(Val285Ala)) and two frameshift variants (c.137delG, p.(Arg46Leufs*32) and c.1239del, p.(Asp413Glufs*42)). In another case report, Iwafuchi et al. (29) detected a stop-gain mutation in the CUL3 gene from a Japanese patient with ASD. This patient was presented with febrile status epilepticus that led to developmental regression, including the loss of verbal ability, eye contact, and ability to do activities of daily living. Exome sequencing revealed a *de novo* double base insertion into the CUL3 gene (c.1758_1759insTG, p. (Thr587*)). Furthermore, Kato et al. (16) reported a novel CUL3 gene mutation (c.158G > a, p.Ser53Asn) that resulted in dysmorphic features, such as macrocephaly, characteristic facial features, and stony skin, in a child with global DD. Most recently, Vincent et al. (30) reported the first case of NDD caused by CUL3 splice site variants. The patient presented with congenital hip dysplasia and global DD. Whole-exome sequencing identified splice site variants in CUL3 (c.379-2A > G). These case reports reveal a pattern of global DD and ID associated with CUL3 *de novo* mutations.

### 4.2. Case–control

Large cohort case–control studies have revealed various *de novo* mutations in CUL3 are associated with NDDs, including ASD, ID, and DD-like phenotypes.

Nonsense and missense mutations of CUL3 have been identified mainly in ASD patients. Severe *de novo* nonsense mutations of CUL3 in ASD were found in 2012 when O’Roak et al. performed exome sequencing on 677 ASD individuals from 209 families (9). Codina-Sola et al. reported CUL3 *de novo* mutations in a cohort of male idiopathic ASD patients (*n* = 36) (31). Larger cohorts were carried out thereafter. De Rubeis et al. analyzed rare coding variants in 3,871 ASD cases and 9,937 ancestry-matched or parental controls, which identified CUL3 as one of 107 high-risk ASD genes that cause *de novo* loss of function (LoF) mutations in more than 5% of ASD (32). Sanders et al. reported CUL3 *de novo* CNVs in patients with ASD (10,220 patients from 2,591 families) in the Simons Simplex Collection (SSC) data (33). The largest array of SPARK analysis findings to date (including 42,607 autism cases) identified CUL3 as a genetic high-confidence LoF variant (36). The latest release of whole-genome sequencing (WGS) data from the Autism Speaks MSSNG resource^1^ identified CUL3 as one of the 134 ASD-associated genes, with a false discovery rate (FDR) of <0.1, in 5,100 ASD and 6,212 non-ASD parents and siblings (37). In the Chinese ASD cohort, Wang et al. conducted a study of recurrent *de novo* and likely gene-disruptive (LGD) mutations (*n* = 1,543), which identified CUL3 *de novo* LGD recurrences (39).

Missense mutations in CUL3 have also been identified in cohorts of ASD combine with ID, DD, or NDDs. Hormozdiari et al. (8) analyzed exome sequencing data from 1,116 individuals with ASD and ID, which identified patients with severe Cul3 missense mutations have more significant intellectual impairment. Wang et al. (38) performed an integrated gene analysis of *de novo* variants in 15,560 ASD and 31,052 DD, identifying CUL3 as one of the *de novo* variance-rich genes in 138 NDDs combinations. In a targeted sequencing study of 208 candidate genes in 11,730 patients and 2,867 controls, CUL3 was found to reach *de novo* significance and identified as one of the 91 NDDs risk genes with ASD and DD biases (34). da Silva Montenegro et al. (35) performed a meta-analysis with more than 20,000 patients from NDDs cohorts and found mutations in CUL3.

Overall, the above evidence supports the notion that CUL3 is a high-risk gene for NDDs, especially ASD.

## 5. Cul3 knockout transgenic animal models

Although Cul3 has been identified as a high-risk ASD gene, the pathophysiological mechanisms underlying the contribution of Cul3 haploinsufficiency to ASD remain largely unknown. This knowledge gap is primarily due to the whole-body deletion of Cul3 resulting in animal death (40), which limits research in this area of brain science.

**Figure 3:**
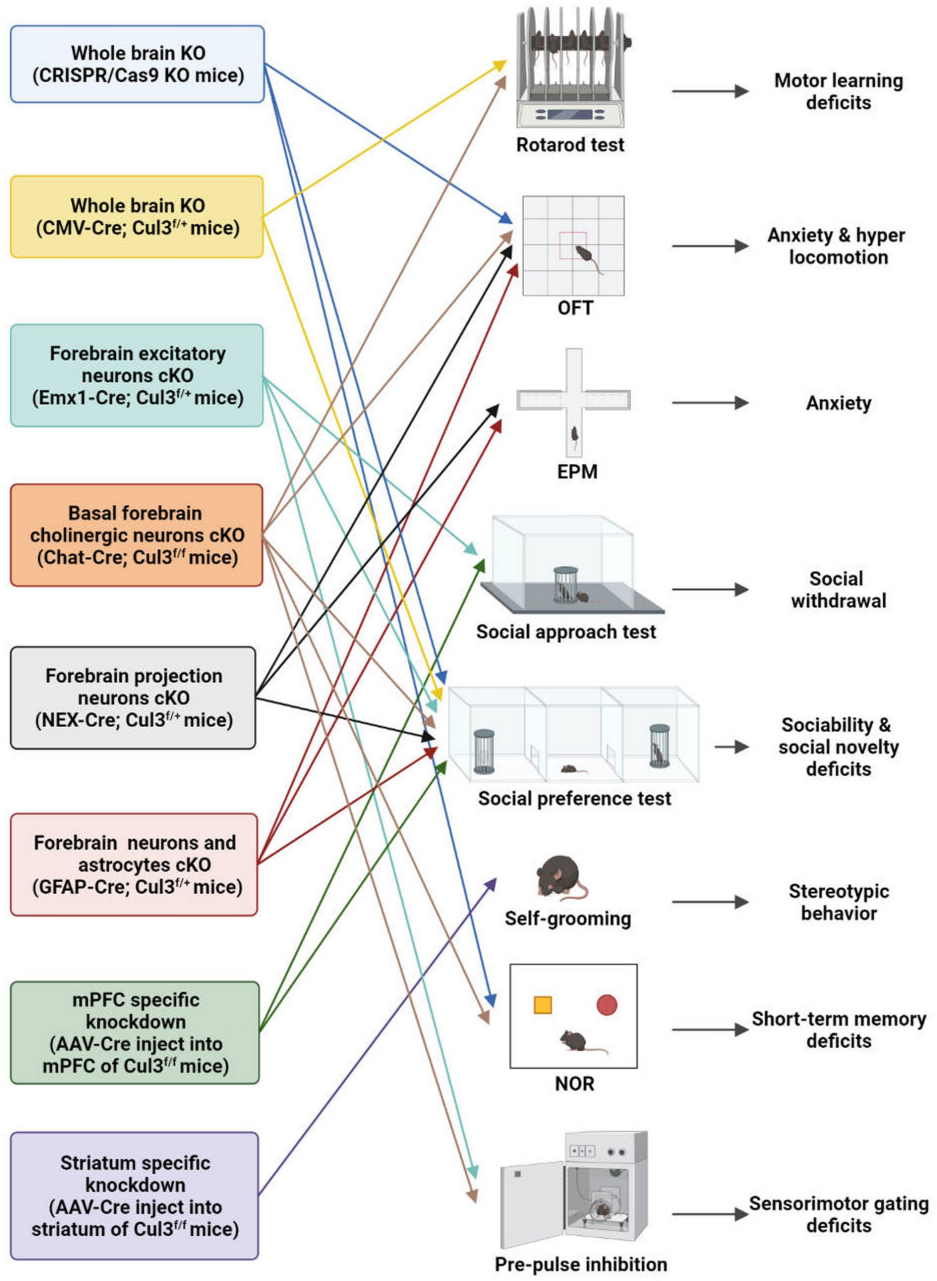
Behavioral characteristics of different Cul3 knockout mouse models. Animal models of ASD with Cul3 knockout (KO) in different brain regions display varying behavioral phenotypes. CRISPR/Cas9 whole brain Cul3-KO mice exhibit hyperactivity, cognitive impairment, and social deficits. CMV-Cre; Cul3^f/+^ whole brain Cul3-KO mice show ASD-like social and cognitive impairments, as well as motor coordination deficits. Mice with Cul3 conditional knockout (cKO) in forebrain excitatory neurons demonstrate ASD-like social and sensory-gating impairment. Mice with Cul3-cKO in basal forebrain cholinergic neurons exhibit ASD-like social and sensory gating phenotypes, along with significant cognitive impairment. Mice with Cul3-cKO in forebrain projection neurons or astrocytes display social interaction disorders and anxiety-like behaviors. mPFC-specific knockdown induces ASD-like social deficits, while striatum-specific knockdown causes repetitive stereotyped behavior. OFT, open field test; EPM, elevated plus mazes; NOR, novel object recognition.

Fortunately, with the advent and newly generated transgenic mice (Cul3^f/f^ and neuron-type specific Cre lines), viral tools, and CRISPR/Cas9 technology, researchers from various institutions worldwide have conducted relevant research on the neurobiological role of Cul3 (Table 2; Figure 3). A variety of Cul3 knockout (KO) and conditional KO (cKO) mouse models have been reported, including those based on Emx1-Cre (25, 26), GFAP-Cre (41), NEX-Cre (41), CMV-Cre (25), Chat-Cre (42), and CRISPR/Cas9 (23).

**Table 2:** Behavioral and electrophysiological features of various Cul3-KO models.

Brain region	Model	Method	Behavior characteristics	Molecular changes	Electrophysiological characteristics
Whole brain	CRISPR/Cas9 KO mice (23)	CRISPR/Cas9 genome engineering	Hyperactivity, cognitive and social impairments.	Reduced dendritic growth, filamentous actin puncta, and spontaneous network activity.	/
	CMV-Cre; Cul3^f/+^ mice (25)	Cul3^f/f^ X CMV-Cre	ASD-like social and motor coordination deficits.	Upregulated Plastin3 leads to defects in neuronal migration.	Reduced sEPSC amplitude and frequency.
Forebrain	Emx1-Cre; Cul3^f/+^ mice (26)	Cul3^f/f^ X Emx1-Cre	ASD-like social and sensory-gating impairment.	Increased RhoA and spine density in mPFC; hypofunction of NMDA receptor; misregulated proteins such as Smyd3.	Reduced amplitude of NMDAR-mediated EPSC.
Hippocampus and cortex	NEX-Cre; Cul3^f/+^ mice & GFAP-Cre; Cul3^f/+^ mice (41)	Cul3^f/f^ X GFAP-Cre or NEX-Cre	Social interaction deficits and anxiety-like behaviors.	Increased eIF4G1 and glutamatergic transmission, disturbed the E-I balance.	Increased mEPSC frequency and AP in CA1 neurons.
Basal forebrain	Chat-Cre; Cul3^f/f^ mice (42)	Cul3^f/f^ X Chat-Cre	ASD-like social, cognitive, sensory-gating, motor coordination deficits, and hyperlocomotion.	/	Reduced frequency of synaptically-driven AP in BF cholinergic neurons.
mPFC	mPFC-specific knockdown (26)	Injected AAV8-CaMKIIα-GFP-Cre into mPFC	ASD-like social deficits.	Hypofunction of NMDA receptor.	Reduced NMDAR-EPSC in mPFC pyramidal neurons.
Striatum	Striatum-specific knockdown (26)	Injected AAV8-CaMKIIα-GFP-Cre into the striatum	Repeat stereotyped behavior.	Attenuated neuronal excitability; decreased D1-MSN excitability.	Reduced AP firing frequency.
Cell culture	CUL3-KO iPSCs (60)	CRISPR/Cas9 genome engineering	/	/	Reduced neuronal excitability, but unaffected basal synaptic transmission.

sEPSC, spontaneous excitatory postsynaptic current; mEPSC, miniature excitatory postsynaptic current; mPFC, medial prefrontal cortex; E/I balance, excitation-inhibition balance; D1-MSN, D1-dopamine receptor-expressing medium spiny neuron; AP, action potential; BF, basal forebrain.

### 5.1. Whole-brain Cul3-KO

To investigate the effects of Cul3 mutations on brain development, Amar et al. (23) employed CRISPR/Cas9 genome engineering technology to create a whole-brain Cul3-KO mouse (Cul3^+/−^)(Table 2; Figure 3). They introduced a 1-bp frameshift insertion into Cul3 exon 6, which is one base pair upstream of the mutated nucleotide in ASD patients, thereby establishing a Cul3^+/−^ haploinsufficiency mouse model on a C57BL/6 N background (9, 23). Cul3^+/−^ mice displayed social and cognitive deficits, as well as hyperactivity, accompanied by reduced dendritic growth and cortical neuronal activity.

Aside from using CRISPR/Cas9 system, another whole-brain Cul3-KO mouse model was obtained by breeding Cul3^f/f^ mice with a CMV-Cre line (B6.C-Tg (CMV-Cre)1Cgn/J) (25). Heterozygous loss of constitutive Cul3 results in several behavioral abnormalities, including social problems, motor dysfunction, and olfactory hyperresponsiveness. However, inducing Cul3 haploinsufficiency after adulthood failed to produce ASD-related behaviors, suggesting an essential role of Cul3 during the critical developmental periods (25).

### 5.2. Cul3-cKO in excitatory neurons of the forebrain and hippocampus

To investigate the forebrain dysfunction found in ASD patients (43–45), Rapanelli et al. crossed Cul3^f/f^ with Emx1-Cre to generate mice with Cul3-cKO in forebrain excitatory neurons (Emx1-Cre;Cul3^f/+^) (26) (Table 2; Figure 3). ASD-like social deficits, including social withdrawal, sociability, and social novelty deficits, as well as sensory gating deficits, were identified in this model, along with NMDA receptor hypofunction and reduced spine density. Additionally, a set of dysregulated proteins, including Smyd3 (a histone methyltransferase involved in gene transcription), were identified in this model.

Dong et al. (41) generated another two mouse models with a hippocampal and cortex-specific Cul3-cKO by crossing Cul3^f/f^ mice with GFAP-Cre or NEX-Cre (NEX-Cre;Cul3^f/+^, GFAP-Cre;Cul3^f/+^)(Table 2; Figure 3). These two models exhibit Cul3 deficiency primarily in excitatory neurons, with GFAP-Cre also targeting astrocytes. Both models display social deficits and an increased anxiety-like phenotype. It was claimed by the author that the GFAP-Cre mouse model expresses Cre mainly in projection neurons in the hippocampus (99%) and cortex (88%) (46, 47). Cul3 homozygous mutation caused by GFAP-driven Cre (GFAP-Cre;Cul3^f/f^) led to reduced cortical thickness, hippocampal deformation, and corpus callosum loss. The heterozygous mutant (GFAP-Cre;Cul3^f/+^) showed increased spine density in pyramidal neurons.

NEX-Cre;Cul3^f/+^ mice exhibit deficits similar to GFAP-Cre;Cul3^f/+^ mice at the behavioral level, but primarily target forebrain projection pyramidal neuron starts at embryonic day 11.5 (48). Additionally, Cul3 deficiency in CA1 neurons in both GFAP-Cre and NEX-Cre lines has profound effects on neuronal and network excitability. It is characterized by an overall E/I imbalance in the CA1 network, manifested as an increased frequency of mEPSC and action potential (AP).

### 5.3. Cul3-cKO in cholinergic neurons of basal forebrain

It is important to note that the above Cul3-cKO mouse model did not reproduce the ID observed in ASD individuals (26). To address this limitation, Rapanelli et al. (42) developed a novel mouse model of Cul3-cKO in cholinergic neurons mainly within the basal forebrain (BF) by breeding Cul3^f/f^ with Chat-Cre (Chat-Cre;Cul3^f/+^) (Table 2; Figure 3). This model recapitulated ASD-like social and sensory gating phenotypes and resulted in significant cognitive impairment, highlighting the importance of BF cholinergic neurons for the cognitive components by projecting to the mPFC. A notable decrease in the occurrence of synaptically-driven, spontaneous action potentials was observed in the cholinergic neurons of the basal forebrain in Chat-Cre;Cul3^f/+^ mice.

### 5.4. Brain region-specific Cul3 knockdown

The relationship between cortico-striatal synaptic changes and symptoms of ASD has been increasingly recognized (49, 50). To investigate this further, Rapanelli et al. (26) employed a brain region-specific Cul3 knockdown (KD) mouse model by injecting AAV8-CaMKIIα-GFP-Cre into the mPFC or striatum of Cul3^f/f^ (Table 2; Figure 3). Their findings revealed that mice with brain region-specific deletion of Cul3 exhibited distinct behavioral deficits. Mice with Cul3-specific KD in the mPFC, a crucial brain region for social cognition and social preference (51–53), displayed social deficits. Meanwhile, Cul3-specific KD in the striatum, an important brain region for habit formation and stereotypic behavior (54–57), led to an increase in repetitive grooming behavior. These results implicate Cul3 as a key molecule responsible for the involvement of these brain regions in distinct behavioral phenotypes.

Moreover, the electrophysiological changes in synaptic function and neuronal excitability caused by region-specific Cul3 deletion are closely related to the behavioral phenotype (26). Specifically, Cul3 deficiency in the mPFC resulted in NMDAR hypofunction, a type of synaptic change frequently found in other genetic models of autism, such as Shank3 or Shank2 deficiency (52, 53, 58) and 16p11.2 mice (59). Cul3 deficiency in the striatum, on the other hand, resulted in reduced AP frequency, especially in putative D1-dopamine receptor-expressing medium spiny neurons (D1-MSN).

### 5.5. Cul3 knockdown in cell culture

The role of Cul3 in pluripotent stem cells (PSCs) has also been investigated beyond animal models (Table 2). Fischer et al. (60) examined this role by using CRISPR/Cas9 to KO Cul3 in human-induced PSCs. The iPSCs were subsequently differentiated into cortical glutamatergic neurons through two distinct methods (small molecule induced or direct induction using lentiviral NGN2 expression). Their study demonstrated that the pluripotency of heterozygous Cul3-KO iPSCs was preserved compared to syngeneic control iPSCs. However, small molecule-mediated differentiation of cortical glutamatergic neurons in Cul3 KO cultures resulted in a significant delay in the transition of proliferating radial glia/NPCs to postmitotic neurons. It also showed reduced neuronal excitability, but unaffected basal synaptic transmission. This identified the crucial role of Cul3 in neuronal differentiation.

## 6. Potential Cul3-involved pathways in NDDs

**Figure 4:**
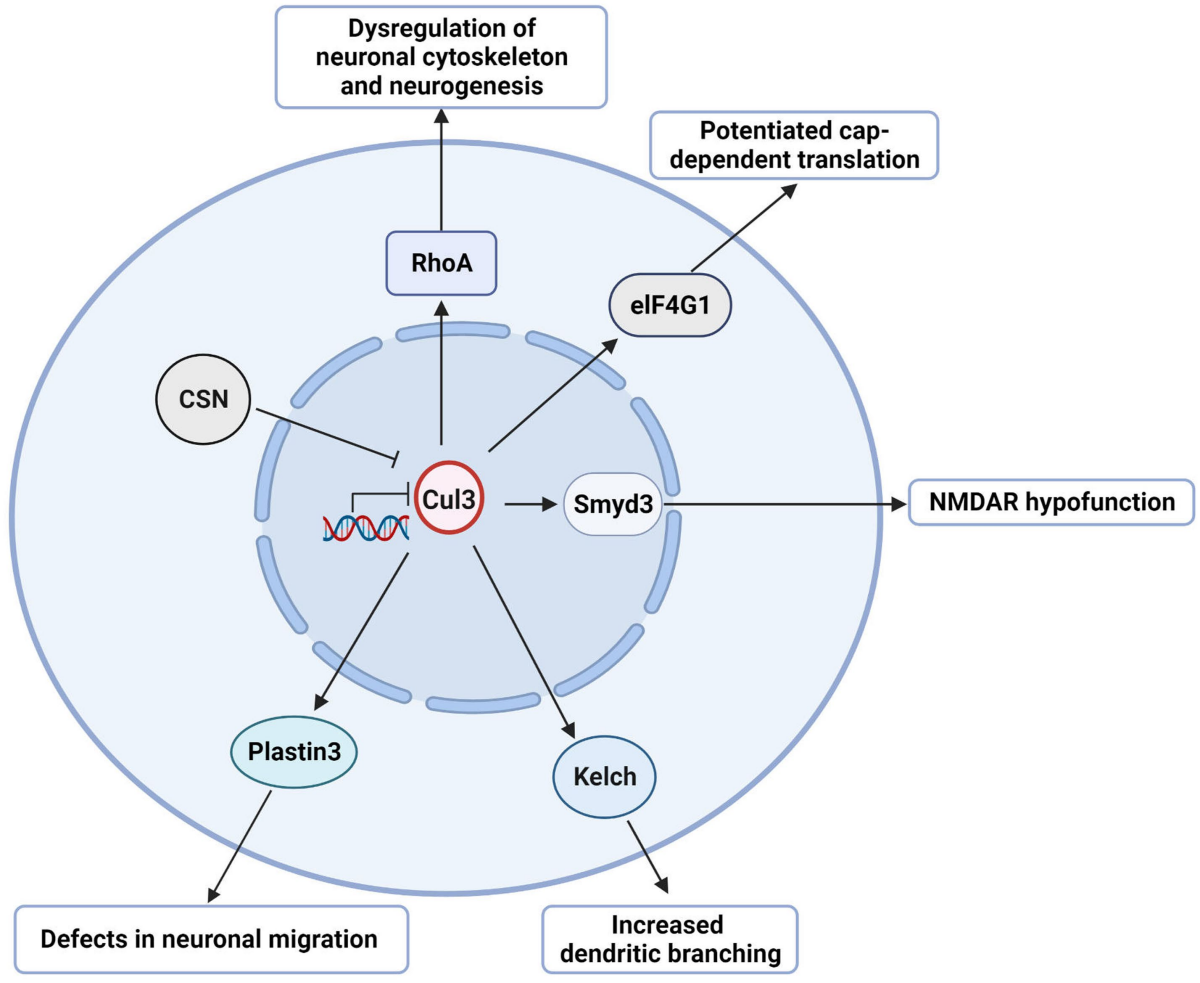
Potential Cul3-involved pathways in NDDs. Schematic illustration of potential Cul3-involved pathways in NDDs. Cul3-KO up-regulates RhoA, leading to dysregulation of the neuronal cytoskeleton and neurogenesis. Cul3 deficiency also up-regulates eIF4G1, promoting cap-dependent translation. Smyd3 also plays as the downstream target of Cul3, which causes NMDAR dysfunction. Additionally, Cul3-KO up-regulates Plastin3, which controls neuronal migration. Furthermore, the COP9 signalosome (CSN) restricts dendritic branching by Cul3-dependent degradation of the actin cross-linked BTB domain protein Kelch. Cul3 deficiency results in Kelch accumulation and uncontrolled dendritic branching. Cul3-KO controls neuronal migration by increasing Plastin3.

As an ASD risk gene, the deficiency of Cul3 can lead to various disturbances, such as cytoskeleton homeostasis, neuronal growth and migration, and dendritic outgrowth. Such disturbances can result in both anatomical and behavioral phenotypes in animal models. However, the molecular mechanisms responsible for Cul3 mutations-related deficits remain unclear. For the following part, we will summarize the potential dysregulated pathways resulting from Cul3 deficiency (Table 3; Figure 4).

**Table 3:** Potential Cul3-involved pathways in NDDs.

Up/Down-stream target	Characteristics	Protein–protein interaction	Potential treatment strategy
RhoA (23)	A substrate of Cul3 ligase which controls neurite outgrowth.	Cul3 deficiency dysregulates neuron cytoskeleton and neurogenesis through increased RhoA levels.	Cortical neurons treated with RhoA inhibitor (Rhosin) showed rescued dendritic length and neural network activity.
eIF4G1 (41)	Cap-dependent translation protein.	Cul3 deficiency increases eIF4G1 to promote cap-dependent translation.	Inhibition (by 4EGI-1) alleviates ASD-associated cellular and behavioral deficits.
Smyd3 (26)	A histone methyltransferase is involved in gene transcription.	Cul3 deficiency upregulates Smyd3 to further inhibit NMDAR function.	Inhibition (by BCI-121) or knockdown of Smyd3 overcomes Cul3 deficient-caused ASD.
COP9 signalosome (CSN) (67)	Member of the conserved 26S proteasome degradation pathway.	CSN restricts dendritic branching *via* Cul3-dependent degradation of the actin cross-linking BTB-domain protein Kelch. Loss of Cul3 and overexpression of Kelch stimulates dendritic branching.	Targeting the CSN-Cul3-Kelch pathway may play a role in intellectual disability and neurodegenerative diseases.
Plastin3 (Pls3) (25)	Involves in neuronal migration.	Cul3 deficiency increases Pls3 and inhibits neuronal migration.	/
Insomniac (Inc) (24)	A putative adaptor of the Cul3 ubiquitin ligase complex.	Cul3 and Inc. are required for rapid ubiquitination of postsynaptic targets and retrograde homeostatic signaling.	Targeting synaptic homeostatic plasticity may be a potential treatment for ASD.

### 6.1. RhoA

One of the top downstream targets of Cul3 could be RhoA, an essential regulator of the actin cytoskeleton, which affects neuronal growth and migration during early brain development (61). It also contributes to neurite outgrowth, whereas upregulated RhoA is associated with loss of spines and reduced neurite outgrowth (61, 62). Cul3 ubiquitinates RhoA and directs its proteasomal degradation (20), suggesting its role in regulating RhoA signaling.

Increased RhoA was found both in whole-brain KO mice (CRISPR/Cas9 Cul3-KO) (23) and forebrain cKO (Emx1-Cre;Cul3^f/+^) (26), which was accompanied by suppressed dendrite length and neurites, reduced total spines, respectively. Administration of Rhosin (23), a pharmacological inhibitor of RhoA, contributes to neurite outgrowth and restores neural network activity caused by Cul3 deficiency.

### 6.2. eIF4G1

Cul3 deficiency impairs the E/I balance and leads to social deficits and anxiety-like behaviors in the forebrain cKO mice (NEX-Cre;Cul3^f/+^, GFAP-Cre;Cul3^f/+^) (41). In this context, Dong et al. (41) identified eukaryotic translation initiation factor 4 gamma 1 (EIF4G1) as a potential target of Cul3 deficiency. Cul3 deficiency results in the upregulation of EIF4G1. Specifically, EIF4G1 serves as a scaffold protein that interacts with RNA helicase EIF4A and cap-binding protein EIF4E at the mRNA 5′ cap to form the eukaryotic translation initiation factor 4F (EIF4F) complex, which initiates cap-dependent protein translation (63, 64). This type of translation is crucial for synaptic transmission and plasticity (65).

Dong et al. (41) further investigated the effect of 4EGI-1, an inhibitor of the EIF4E-eIF4G1 complex, which ameliorated deficits in glutamate release, spine density, E/I balance, and social interaction in Cul3-deficient mice. These results suggest that Cul3 plays a vital role in neural development and synaptic transmission by regulating EIF4E-eIF4G1-dependent translation.

### 6.3. Smyd3

From the large-scale proteomics analysis, SET and MYN-domain containing protein 3 (Smyd3), a histone methyltransferase involved in gene transcription, was identified as a Cul3-deficient regulator in the forebrain-cKO model (Emx1-Cre; Cul3^f/+^) (26). Histone methylation is a well-known histone modification that plays a significant role in regulating gene activity (66). Smyd3 is a chromatin modifier which reported to regulate trimethylate histone 3 lysine 4 (H3K4) that promotes transcription (66).

Upregulation of Smyd3 was found in Emx1-Cre; Cul3^f/+^ (26). Inhibition (BCI-121, a specific Smyd3 inhibitor) or knockdown of Smyd3 improved social deficits and restored NMDAR function in the mPFC of forebrain Cul3-deficient mice (26). Therefore, Smyd3 may be a potential therapeutic target for Cul3-related autism.

### 6.4. CSN-Cul3-Kelch pathway

A study reported that the COP9 signalosome (CSN) restricts dendritic branching *via* the Cul3-dependent degradation of the actin-crosslinked BTB domain protein Kelch (67). The CSN complex comprises eight conserved proteins and acts as a master regulator of Cullin-RING-based ubiquitin ligases (68). In Drosophila, CSN has been identified as a key regulator of dendritic morphogenesis (67). Specifically, CSN stimulates dendritic arborization through Cullin1-mediated proteolysis.

Djagaeva et al. (67) found that CSN inhibits dendritic branching by controlling the Cul3 function. Loss of Cul3 leads to excessive dendritic branching, while the actin-crosslinked BTB domain protein Kelch is a downstream target of Cul3-dependent neuronal degradation. Kelch accumulation due to impaired Cul3-dependent turnover or ectopic expression results in uncontrolled dendritic branching. These findings suggest that the CSN pathway regulates neuronal networks in a multi-layered manner by controlling Cul3, providing insight into the potential role of the CSN-Cul3-Kelch pathway in NDDs.

### 6.5. Plastin3

Recent studies have demonstrated that during brain development, Cul3 regulates neuronal migration, which is crucial for the precise assembly of the cerebral cortex (25). Cul3 plays a role in controlling the abundance of cytoskeleton and adhesion proteins in mouse embryos. Notably, Cul3 deficiency inhibits neuronal migration by increasing Plastin3 (Pls3), a novel molecule that exhibits an inverse relationship between its concentration and the rate of neuronal migration (25). This indicates that drugs capable of reducing Pls3 activity may have therapeutic potential for patients with CUL3-related conditions.

## 7. Perspective

Overall, recent progress in understanding Cul3’s role in brain development has identified it as a risk gene for NDDs, particularly ASD, through large-scale sequencing. Multiple transgenic animal models such as whole-brain KO, neuronal type-specific cKO, and region-specific KD have been developed to investigate behavioral characteristics. Several downstream pathways have been identified as potential targets for treating Cul3 deficiency-induced NDDs.

CUL3’s widespread expression throughout the brain during development suggests its high correlation with NDDs. High-throughput data from the human brain reveals that CUL3 is highly expressed during fetal development and adolescence. Which is further validated in both humans and mice at the protein and mRNA levels. This indicates that CUL3 plays an important role in early brain development and may also be involved in postnatal psychiatric disorders. Therefore, characterizing the biology of CUL3’s high expression during adolescence would be of scientific interest as it could provide valuable insights into the role of CUL3 in adolescents.

CUL3’s extensive expression in the brain during development suggests its strong correlation with NDDs. High-throughput data from the human brain (27) indicates that CUL3 is highly expressed during fetal development and adolescence, which has been verified in both humans and mice at the protein and mRNA levels (25). This suggests that CUL3 plays a significant role in early brain development and may also be implicated in postnatal psychiatric disorders. Therefore, characterizing the biology of CUL3’s high expression during adolescence could provide valuable insights into its role in adolescent development.

Controversial results have been found in adulthood Cul3 deficiency. Animals with Cul3 KD in the mPFC and striatum (26) induced by AAV injection exhibited social deficits and repetitive behaviors, respectively. However, induction of Cul3 haploinsufficiency at P30 (tamoxifen injected at P30 in Cag-CreER;Cul3^f/+^ mice) did not result in any major ASD-like behavioral defect (25). These differences may be due to the different methods employed, as tamoxifen injection induces Cre expression only within a time window of a few hours, while AAV induces long-lasting Cre expression over several months. Therefore, further exploration of multiple days of tamoxifen-induced Cul3 KO during adulthood is warranted.

It is noteworthy that various techniques have revealed different levels of Cul3 expression in the hippocampus. High-throughput sequencing of hippocampal tissue has been utilized to determine average Cul3 mRNA expression (Figure 2C). Conversely, higher levels of Cul3 have been found in the pyramidal and granule cell layers of CA1 and DG of the hippocampus (Figure 2D) using *in-situ* RNA staining, which provides spatial distribution information. These differences may be attributed to the different methods applied. The results of high-throughput sequencing are subject to mRNA extraction from brain tissue, which can be affected by the total volume of the brain region. Therefore, the results represent an average expression level. In contrast, *in-situ* hybridization is a more accurate method as it is susceptible to cell numbers, providing layer-specific information. To further address the brain region and layer-specific differences of Cul3, cutting-edge spatial transcriptome techniques such as MERFISH (69) and stereo-sequence (70) would provide useful detailed information for Cul3-related studies.

To explore Cul3 deficiency in NDDs, different animal models have been generated. In accordance with the brain-wide expression, two types of whole-brain Cul3-KO models have been used: CRISPR/Cas9 line (23) and CMV-Cre;Cul3^f/+^ (25). As a relatively higher expression of Cul3 is observed in the isocortex and hippocampus, two lines of forebrain Cul3-cKO models have been created: Emx1-Cre;Cul3^f/+^ (26) and NEX-Cre;Cul3^f/+^ (41). However, animal models targeting the cerebellum are still missing, although high expression of Cul3 has been found in this region, both from high-throughput sequencing and *in-situ* hybridization. Notably, significantly higher expression of Cul3 is observed mainly in glutamatergic and inhibitory neurons in the adult human brain (25). To date, no interneuron-specific Cul3-cKO models, such as PV, SST, or VIP interneurons, have been reported. Although researchers have targeted the striatum with Cul3-KD and found it to be more susceptible in D1-MSNs (26), no cell-type specific cKO experiments have been carried out within the striatum, especially cKO in D1-MSN or D2-MSN, which require further investigation. In non-neuronal cell types, only astrocytes (GFAP-Cre;Cul3^f/+^) (41) have been mentioned but not fully explored. Other cells such as glial cells and microglia have not been reported and require further investigation. The development of new neuron-specific and non-neuron-specific Cul3-cKO mouse models will aid in further understanding the mechanism of Cul3 as a high-risk gene for ASD in different cell types.

Multiple studies have reported that Cul3 haploinsufficiency induces neuronal functional alterations. For example, Morandell et al. (25) discovered reduced spontaneous activity in layer 2/3 excitatory neurons of the somatosensory cortex in whole-brain Cul3-KO mice (CMV-Cre;Cul3^f/+^), while Rapanelli et al. (26) observed a decreased amplitude of NMDAR-mediated EPSC in layer 5 excitatory neurons of the mPFC in forebrain Cul3-cKO mice (Emx1-Cre;Cul3^f/+^). In contrast, Dong et al. (41) found increased mEPSC frequency and AP in CA1 pyramidal neurons in GFAP-Cre;Cul3^f/+^ and NEX-Cre;Cul3^f/+^ mice. These discrepancies might be attributed to the diverse brain regions analyzed (cortex versus hippocampus) or the different transgenic lines employed. These findings support the E/I imbalance hypothesis of ASD and suggest that Cul3 may have distinct functions in neurons across different brain regions.

Moreover, only one study has examined circuit changes and reported that the activation of BF cholinergic neurons projecting to the mPFC can reverse cognitive deficits in Chat-Cre;Cul3^f/+^ mice (42). More research is needed to investigate long-distance circuits. Combining Cul3-cKO in various brain regions with optogenetics or chemogenetics can help uncover the potential circuits underlying ASD abnormalities, such as cortex➔striatum, cortex➔ thalamus, and hippocampus➔striatum. In addition, micro-circuit studies are lacking, and multi-patch would be useful to investigate the micro-circuit changes while using Cul3-cKO in specific neuronal types or whole-brain Cul3-KO.

To investigate potential downstream molecular targets, most studies have employed proteomics (23, 25, 41) and RNA-seq (23). These methods have allowed for the screening of several targets, including RhoA (23), Smyd3 (26), and EIF4 (41). However, as Cul3 directly modulates protein degradation, any resulting transcriptional changes would be indirect in nature. Therefore, future studies should pay closer attention to RNA-seq data to determine whether compensatory effects are present. Additionally, since Smyd3 and EIF4 are involved in H3K4 modulation and cap-dependent protein translation, respectively, other high-throughput techniques, such as ATAC-seq, may be useful in identifying any epigenetic changes.

## Author contributions

PL conducted the literature search and drafted the paper. JY, SW, TY, WZ, and WW wrote part of the paper. TT designed the outline of the review, supervised the entire process, and revised the paper. All authors contributed to the article and approved the submitted version.

## Funding

This work was supported by Science Foundation of Oujiang Laboratory to TT (OJQD2022002).

## Conflict of interest

The authors declare that the research was conducted in the absence of any commercial or financial relationships that could be construed as a potential conflict of interest.

## Publisher’s note

All claims expressed in this article are solely those of the authors and do not necessarily represent those of their affiliated organizations, or those of the publisher, the editors and the reviewers. Any product that may be evaluated in this article, or claim that may be made by its manufacturer, is not guaranteed or endorsed by the publisher.

## Footnotes

^1^ https://research.mss.ng
